# Understanding sexual violence and factors related to police outcomes

**DOI:** 10.3389/fpsyg.2022.977318

**Published:** 2022-09-01

**Authors:** Kari Davies, Ruth Spence, Emma Cummings, Maria Cross, Miranda A. H. Horvath

**Affiliations:** ^1^Department of Psychology, Bournemouth University, Poole, United Kingdom; ^2^Centre for Abuse and Trauma Studies, Middlesex University London, London, United Kingdom; ^3^Department of Psychology, Carleton University, Ottawa, ON, Canada; ^4^Institute for Social Justice & Crime, University of Suffolk, Ipswich, United Kingdom

**Keywords:** sexual violence, rape, rape myths and stereotypes, policing, police outcomes

## Abstract

In the year ending March 2020, an estimated 773,000 people in England and Wales were sexually assaulted. These types of crimes have lasting effects on victims’ mental health, including depression, anxiety, and post-traumatic stress disorder. There is a large body of literature which identifies several factors associated with the likelihood of the victim reporting a sexual assault to the police, and these differences may be due to rape myth stereotypes which perpetuate the belief that rape is only “real” under certain conditions. Less is known, however, about the effect these rape myths and stereotypes have on the investigation process itself and the subsequent police outcomes assigned to sex offences. This study aimed to address this gap, providing a profile of all RASSO (rape and serious sexual offences) committed over a 3-year period in one English police force, the police outcomes of these offences, and whether any offences, suspect, or victim variables were associated with different outcomes, in particular the decision to charge or cases where victims decline to prosecute. In line with previous research, the majority of victims were female while the majority of suspects were male, and the most frequent victim–suspect relationship was acquaintance, followed by partner/ex-partner. Charge outcomes were more likely in SSOs and less in rape offences, more likely with stranger offences and less likely than offences committed by partners/ex-partners and relatives, and some non-white suspects were more likely to be charged than suspects of other ethnicities, including white suspects. Victim attrition was more likely in cases where the suspect was a partner or ex-partner and least likely where the suspect was a stranger, more likely in SSOs than in rape cases, and more likely when the victim ethnicity was “other”. Law enforcement should be aware of the potential biases, both relating to rape myths and stereotypes and to the biased treatment of victims and suspects based on demographic characteristics, and work to eliminate these to ensure a fairer and more effective RASSO investigative process.

## Introduction

Reports of sexual violence, including rape and serious sexual offences (RASSO), have steadily increased over the last few years, with reported numbers of cases at an all-time high in England and Wales (Home Office, 2022). RASSO can have lasting and devastating effects on victims^1^ in several ways including extreme and incessant self-blame (Moor, 2007). Many victims experience the police process as “secondary victimisation,” which exacerbates trauma (Campbell and Raja, 1999), with victims facing blame and disbelief from the very people responsible for investigating these offences (Wager et al., 2021). Victims are at risk of developing myriad mental health disorders (Oshodi et al., 2020), including an increase in psychological disorders after the offence, such as major depressive disorder (Petrak et al., 1997), post-traumatic stress disorder (Epstein et al., 1998), and anxiety disorders (Petrak et al., 1997). As well as poor mental health outcomes, victims may also suffer from the physical effects of sexual assault, such as contracting sexually transmitted infections and becoming pregnant, as well as the bodily harm they may have sustained at the time of the assault (Cybulska, 2007; Linden, 2011).

The increase of reporting RASSO has been attributed to the increased spotlight on sex offences, such as the #metoo movement and several high-profile sex offences cases reported in the media (BBC, 2020; Levy and Mattsson, 2021). Despite this reporting increase, charge and conviction rates remain low, with only 1.6% of rapes reported to the police in 2020 resulting in someone being charged (HM Government, 2021). Victim attrition (where a victim withdraws support for the progression of a criminal investigation, as opposed to the formal retraction of an allegation; HMIC/HMCPSI, 2012) in RASSO investigations is high, with large numbers of victims withdrawing from the investigation process before a decision whether or not to charge can be made (Hohl and Stanko, 2015). It is also important to note that some groups still remain underrepresented within the criminal justice system, suggesting that some victims are more likely to seek a police response in the first place (Walker et al., 2021). Despite a number of reviews, little seems to be able to be done in terms of improving the charge rates associated with this offence type that has an already low reporting rate (Stern, 2010).

### Rape myth stereotypes and their effect on reporting and policing outcomes

Rape mythology has been in discussion since the 1970s (Brownmiller, 1975), including concerns that rape myths reinforce notions of what sexual assault is (and what it is not) and who a credible victim is (Brownmiller, 1975). Burt (1980, p. 217) defined rape myths as “prejudicial, stereotyped and false beliefs about rape, rape victims and rapists.” This definition still resonates with the beliefs and concerns surrounding rape myths in today’s society. Of a randomly sampled survey of 1,095 adults from across England and Wales, over one quarter believed a woman is partially or completely responsible for being raped if she wore revealing clothing, and over one third believed she was at least partially responsible if she behaved flirtatiously (Amnesty International UK, 2005). Several tools have been designed to measure rape myth acceptance, such as the Illinois Rape Myth Acceptance scale (IRMA; Payne et al., 1999), and the Acceptance of Modern Myths about Sexual Aggression scale (AMMSA; Gerger et al., 2007), with research being conducted to update these tools to reflect, for instance, the subtleties of rape myths (McMahon and Farmer, 2011). Cultural differences in the belief of rape myths have also been explored (Stephens et al., 2016), as well as myths relating to specific circumstances such as domestic violence (Peters, 2008; Giger et al., 2017; Lelaurain et al., 2019). There are factors that have been associated with the increased belief in rape myths, such as being male (Hammond et al., 2011), demonstrating hostility toward women (Lonsway and Fitzgerald, 1995), greater Just World Belief (Russell and Hand, 2017), and having less quality contact with counter-stereotypical women (Taschler and West, 2017), as well as other factors such as greater alcohol consumption, greater religiosity, and lower GPA scores (Navarro and Tewksbury, 2017). While there is some evidence to suggest that rape myth acceptance can be decreased by attending rape awareness workshops (Hinck and Thomas, 1999), other evidence suggests the rape myths are enduring and resistant to training designed to combat these attitudes, with behavioural performance, but not cognitive or attitudinal outcomes, changing after training (Lonsway et al., 2001). While certain beliefs about what rape “should” be are widespread and feed into the idea of a “real rape stereotype,” they are not supported by ongoing research into the area. For instance, it is commonly thought that rape is most often perpetrated by an unknown individual, but it is evident that rape and sexual assault is more often committed by a known offender (Waterhouse et al., 2016).

There is evidence to suggest that these erroneous views about rape have an effect on a victim’s decision to report an offence to the police. Despite the greater prevalence of acquaintance rape, for instance, a stranger rape is more likely to be reported to the police than an acquaintance rape (Campbell et al., 2001). Wolitzky-Taylor et al. (2011) found similar barriers to reporting, such as the suspect not being a stranger, or the victim using drugs or alcohol, which demonstrates many of the same barriers faced by victims for decades and the influence of “real rape” stereotyping on the decision to report (Jordan, 2011). Reasons given for not reporting a rape to the police included *embarrassment* (over 40%) and *didn’t think they would believe me* (over 20%) (Office for National Statistics, 2021), which could suggest that victims are aware of the prevalence of disbelief in law enforcement regarding rape offences and are therefore deterred from reporting, particularly when their offence does not adhere to the “real rape” stereotype (Smith and Daly, 2020; Champion et al., 2021).

These biases can also be seen to affect, not just the decision to report, but the investigative process and police outcomes should a victim decide to disclose the offence to the police (Murphy et al., 2022). Indeed “In terms of the investigation and prosecution of rape offences, such myths are influential from the moment of first reporting and the initial response the complainant receives, right through to decisions regarding whether or not an offender will be prosecuted, and whether courts and juries will decide to convict or acquit.” (Jordan, 2011, p. 243). Sleath and Bull (2015), for instance, found that United Kingdom police officers were more likely to subscribe to *she lied* myths than a control group of United Kingdom students, and there is evidence to suggest officers substantially overestimate the number of false allegations (Rumney, 2006; Saunders, 2012; McMillan, 2018), based on “stereotypes regarding the complainant’s behaviour, attitude, demeanour or possible motive” (Jordan, 2004, p. 48). Hansen et al. (2019), having reviewed 248 rape cases reported to a single Danish police district, found no evidence of investigative bias toward rape stereotypes but, using a combination of four case characteristics associated with common rape myths (namely whether the perpetrator was a stranger, victim intoxication, evidence of victim resistance, and the infliction of physical injuries to the victim), significantly predicted the likelihood of cases continuing for prosecution, with Campbell et al. (2001) also demonstrating cases involving a stranger offender being more likely to lead to prosecution. Further, in reviewing 679 cases reported to a single United Kingdom police force during a 2-month period, Hohl and Stanko (2015) presented evidence to support the influence of some rape myths on case attrition, such as victim alcohol consumption prior to the incident, but not the involvement of physical injury to the victim nor the victim-perpetrator relationship, suggesting that these stereotypes affect a range of different investigative outcomes. In a study directly investigating the relationship between rape myth acceptance and the judgements police officers make about victim and suspect responsibility and the authenticity of the report, it was found that higher rape myth acceptance in officers was associated with victims being held more responsible for the offences and suspects less responsible, with the case being considered less “authentic” overall (Hine and Murphy, 2019). While not the primary focus of this paper, it is also worth noting that post policing outcome these myths are likely to affect the latter parts of the criminal justice process, such as juror decision making (Willmott et al., 2021). In summary, there is some evidence to suggest that these widely held and erroneous beliefs not only deter victims from reporting but, if they do decide to report, may result in them being met with bias from law enforcement which affects the progress of the investigation.

### The effect of poor policing on victim wellbeing

While literature on this topic is mixed (Sleath and Bull, 2017), likely owing to difficulties isolating the influence of rape myths from other factors such as evidentiary value of physical injuries (Hansen et al., 2019) and geographical differences associated with different local cultures and training provision, the results of these biases within the criminal justice system are that, as well as deterring victims from reporting offences in the first place, rape myths may also have an effect on the process of a police investigation and the subsequent police outcome. Not only does this impact on a victim’s right to justice, but it can have a direct impact on a victim’s wellbeing. Going through the process of reporting and pursuing a rape offence can be a traumatising experience for the victim (Maier, 2008), with evidence to suggest that the legal process associated with reporting and pursuing a rape case can negatively impact a victim (Sloan, 1995; Campbell and Raja, 1999), and there is evidence to suggest that poor policing can make the victim’s experience even worse. For instance, while 52% of victims rated the experience of seeking legal aid harmful, cases were more likely to be rated as harmful if the case did not result in a prosecution (Campbell et al., 2001). Thus, understanding how stereotypical beliefs may affect the investigative process in terms of the outcomes assigned to cases is important, both from a justice but also from a victim welfare perspective.

### The current study

Access to a large policing dataset as part of Project Bluestone^2^ provided the researchers with an opportunity to explore the nature of sexual violence and the criminal justice process by analysing over 10,000 RASSO reports from a 3-year period in one English police force. Importantly, the dataset allowed us to provide a more complete picture of the types of offences being reported, because we are considering all “incidents” reported within this police force, rather than only convicted cases. The data also enabled us to assess whether certain policing outcomes, such as the closing of a case based on insufficient evidence, or the progression of a case to charge, is associated with any of the victim or suspect details contained within the data. If differences were seen in outcomes, they were assessed to explore whether the rape myth narrative influences the criminal justice process, not just at the reporting stage, but also at the investigation and charging stage.

In summary, the aims of this study were to explore:

1. The types of RASSO offences reported, and the number and types of suspects and victims associated with these offences;
2. The outcomes of RASSO;
3. Whether any specific variables are associated with particular outcomes, and whether this fits with rape myths and stereotyping.

## Materials and methods

### Sample

All incidents of RASSO recorded by Avon and Somerset Constabulary (ASC), United Kingdom, during a 3-year period from the beginning of January 2018 to the end of December 2020 were sampled. The types of offences included in the RASSO definition – as defined by ASC – are listed in Supplementary Appendix A. This totalled 10,348 offences,^3^ including 8,273 unique victim identities and 6,010 unique suspect^4^ identities. 10,040 of these offences were associated with different suspect and victim identities, meaning that in 308 offences either multiple suspects or multiple victims offending/being offended against during the same incident. The dataset contained 47 variables which related to the offence itself, as well as the suspects and victims involved in the offences.

### Procedure

Data were collected as part of Project Bluestone, taken from records held by ASC in their crime recording system, NICHE. NICHE is a system used by officers and administrative staff in ASC that records details of every offence reported to them, including information on the crime itself, the people involved, and the process of the investigation and police outcomes assigned. ASC’s NICHE system is not linked to other police forces’ systems in the United Kingdom and therefore contains details of offences committed in their jurisdiction only. Variables the researchers were interested in exploring as part of Project Bluestone were requested from ASC; the data were then collated by a member of ASC, who ensured that all data were fully anonymised before securely transferring it to the research team. The data then underwent some recoding before it could be analysed:

• Ethnicity was grouped into white, dark European, African Caribbean, Asian, Chinese, Japanese or other Southeast Asian, Arabic, and other using police IC codes; unknown ethnicity was excluded from analysis;
• The 20 relationship options (see Supplementary Appendix B for a full list) were grouped into relative, partner, acquaintance, and stranger; unknown relationships were excluded from analysis^5^ ;
• Any suspect or victim gender that was marked as “indeterminate” was excluded from the analysis due to low numbers;
• The victim age, both at reporting and when the offence occurred, was grouped into 16 years old and over, 13--15 years old, and under 13 years old^6^ ;
• The suspect’s age, both at reporting and when the offence occurred, was grouped into 16 years old and over, 13–15 years old, and under 13 years old;
• RASSO was grouped into all rape offences, all sexual assault offences, and all non-contact offences;
• The police outcomes were also recoded, from 21 outcomes (see Supplementary Appendix C for a full list) into five, broader categories: (1) case not progressed for logistical reasons (such as the suspect dying); (2) NFA (no further action); (3) case is resolved but not charged (such as the use of a community resolution); (4) the victim declines to prosecute; and (5) suspect charged.

### Analysis

Descriptive data are presented for the victims and suspects. Chi-square analysis was used to determine any significant differences between offence type and victim–suspect relationship, and offence type and outcome. One-way ANOVAs with type of offence as the factor and age entered as the dependent variable were used to assess mean age differences. To establish relationships between victim and suspect demographics and an outcome of charged or an outcome of victim does not want to proceed, univariate logistic regressions and multiple binary logistic regressions using a backward stepwise elimination procedure were conducted. Type of offence, victim–suspect relationship, victim and suspect sex, victim and suspect age, and victim and suspect ethnicity were entered as predictors and either “charged yes/no” or “victim does not want to proceed yes/no” were entered as the outcome. All analyses were performed using SPSS version 28.

## Results

### Types of rape and serious sexual offences offences reported

There were 4,957 (47.90%) rapes, 5,357 (51.77%) SSOs, and 34 (0.33%) non-contact offences.

### Victims

There were 10,348 RASSO occurrences for 2018–2020; which included 8,273 victims.

#### Victim age

Each victim had their age recorded for when the offence was committed and for when the offence was reported. Cases where the age was missing or below 0 were excluded. The average age at the time of offence was 24.66 years (SD: 14.67; range: 0–116; missing = 831, 8.22%, plus another 59 cases (0.59%) where age was under 0) and average at the time of reporting was 27.80 [SD: 14.89; range: 0–100; missing = 169 (1.68%), plus another 3 cases (0.00%) where age was under 0]. Table 1 shows the victim age when offences were committed and when they were reported, overall and broken down by offence type. Non-contact victims were significantly younger than SSO and rape victims and SSO victims were significantly younger than rape victims at the time of the offence [*F*(2, 9,147) = 56.09, *p* < 0.001] and at time of reporting [*F*(2, 9,865) = 61.08, *p* < 0.001].

**TABLE 1:** Victim age when offences were committed and when they were reported, overall and broken down by offence type.

	Age when reported				Age when committed			
		
	Rape	SSO	Non-contact	All offences	Rape	SSO	Non-contact	All offences
Under 13	202 (4.22)	677 (13.40)	18 (60.00)	897 (9.09)	491 (11.19)	1,047 (22.10)	24 (92.31)	1,562 (17.07)
13-15	416 (8.69)	656 (12.98)	3 (10.00)	1,075 (10.89)	471 (10.74)	653 (13.79)	–	1,124 (12.28)
16+	4,167 (87.08)	3,720 (73.62)	9 (30.00)	7,896 (80.02)	3,425 (78.07)	3,037 (64.11)	2 (7.69)	6,464 (70.64)
**Total**	**4,785 (100)**	**5,053 (100)**	**30 (100)**	**9,868 (100)**	**4,387 (100)**	**4,737 (100)**	**26 (100)**	**9,150 (100)**

#### Victim gender

Gender was missing in 256 (2.47%) instances. Table 2 shows that females were the majority of victims for all types of offence, however, this was most noticeable for rape, where male victims made up less than 10% of cases.

**TABLE 2:** The gender of the victims in the dataset.

	Rape	SSO	Non-contact	Total (%)
Male	428 (8.79)	933 (17.98)	11 (34.38)	1,372 (13.59)
Female	4,442 (91.21)	4,257 (82.02)	21 (65.63)	8,720 (86.41)
**Total**	**4,870 (100)**	**5,190 (100)**	**32 (100)**	**10,092 (100)**

#### Victim ethnicity

Ethnicity was missing for 4,623 (44.68%) cases. The majority of victims were white for all offence types (see Table 3).

**TABLE 3:** Victim ethnicity broken down by offence type.

	Rape	SSO	Non-contact	Total (%)
IC1 – White	2,744 (87.28)	2,300 (89.46)	10 (100)	5,054 (88.28)
IC2 – Dark European	79 (2.51)	51 (1.98)	–	130 (2.27)
IC3 – African Caribbean	142 (4.52)	87 (3.38)	–	229 (4.00)
IC4 – Asian	51 (1.62)	50 (1.94)	–	101 (1.76)
IC5 – Chinese, Japanese, or other Southeast Asian	16 (0.51)	7 (0.27)	–	23 (0.40)
IC6 – Arabic	9 (0.29)	4 (0.16)	–	13 (0.23)
Other	103 (3.28)	72 (2.80)	–	175 (3.06)
**Total**	**3,144 (100)**	**2,571 (100)**	**10 (100)**	**5,725 (100)**

### Suspects

There were 10,348 RASSO occurrences for 2018–2020; of these 3,317 (32.05%) had no identified suspect, whereas 7,031 (67.95%) had at least one suspect identified.

#### Suspect age

Each suspect had their age recorded for when the offence was committed and for when the offence was reported. Cases where the age was missing or below zero were excluded. Average age at the time of offence was 32.59 [SD: 16.13; range: 0–100; missing = 4,042, 39.06%, plus another 3 cases (0.00%) where age was under 0] and average at the time of reporting was 35.84 [SD: 17.83; range: 0–120; missing = 3,636 (35.14%), plus another 1 case (0.00%) where age was under 0]. Table 4 shows the suspect age when offences were committed and when they were reported, overall and broken down by offence type. SSO suspects were significantly older than rape suspects at the time of offence, [*F*(2, 6,300) = 20.85, *p* < 0.001], and this was the same for age at reporting [*F*(2, 6,710) = 20.79, *p* < 0.001]. There were no significant age differences with non-contact suspects and rape/SSO suspects.

**TABLE 4:** Suspect age when offences were committed and when they were reported, broken down by offence type.

	Age when reported				Age when committed			
		
	Rape	SSO	Non-contact	Total	Rape	SSO	Non-contact	Total
Under 13	78 (2.52)	183 (5.73)	1 (5.88)	262 (4.16)	31 (0.93)	130 (3.85)	–	161 (2.40)
13-15	243 (7.85)	356 (11.15)	1 (5.88)	600 (9.52)	186 (5.61)	333 (9.87)	1 (5.26)	520 (7.75)
16+	2,773 (89.63)	2,653 (83.11)	15 (88.24)	5,441 (86.32)	3,101 (93.46)	2,911 (86.28)	18 (94.74)	6,030 (89.85)
**Total**	**3,094 (100)**	**3,192 (100)**	**17 (100)**	**6,303 (100)**	**3,318 (100)**	**3,374 (100)**	**19 (100)**	**6,711 (100)**

#### Suspect gender

Suspect gender was missing in 3,375 instances (32.61%). The majority of suspects were male in rape, SSO, and non-contact offences as can be seen in Table 5.

**TABLE 5:** Suspect gender, broken down by offence type.

	Rape	SSO	Non-contact	Total (%)
Male	3,395 (99.27)	3,248 (91.91)	18 (94.74)	6,661 (95.53)
Female	25 (0.73)	286 (8.09)	1 (5.26)	312 (4.47)
**Total**	**3,420 (100)**	**3,534 (100)**	**(100)**	**6,973 (100)**

#### Suspect ethnicity

Suspect ethnicity was missing in 5,416 instances (52.33%). The majority of suspects in all offence types were white as shown in Table 6.

**TABLE 6:** Suspect ethnicity, broken down by offence type.

	Rape	SSO	Non-contact	Total (%)
IC1 – White	2,063 (80.09)	1,931 (82.45)	13 (92.86)	4,007 (81.24)
IC2 – Dark European	82 (3.18)	67 (2.86)	–	149 (3.02)
IC3 – African Caribbean	261 (10.13)	157 (6.70)	–	418 (8.48)
IC4 – Asian	92 (3.57)	96 (4.10)	1 (7.14)	189 (3.83)
IC5 – Chinese, Japanese, or other Southeast Asian	7 (0.27)	7 (0.30)	–	14 (0.28)
IC6 – Arabic	17 (0.66)	19 (0.81)	–	36 (0.73)
Other	54 (2.10)	65 (2.78)	–	119 (2.41)
**Total**	**2,576 (100)**	**2,342 (100)**	**14 (100)**	**4,932 (100)**

#### Suspect–victim relationship

Relationship data were missing in 5,427 (52.44%) of cases. Table 7 shows that where these data available, the most common relationship was acquaintance, and almost a quarter of cases were committed by (ex-) partners. Differences between relationships by offence type were significant (*x*^2^ = 620.90, *p* < 0.001). Partners were more likely to commit rape and less likely to commit SSO than any other relationship. Acquaintances were least likely to commit non-contact offences and more likely to commit rape than strangers, whilst strangers were more likely to commit SSO than acquaintances.

**TABLE 7:** The suspect–victim relationship, broken down by offence type.

	Rape	SSO	Non-contact	Total (%)
Partner	948 (39.98)	257 (10.16)	–	1,205 (24.49)
Relative	305 (12.86)	513 (20.28)	9 (45.00)	827 (16.81)
Acquaintance	850 (35.85)	1,238 (48.93)	4 (20.00)	2,092 (42.51)
Stranger	268 (11.30)	522 (20.63)	7 (35.00)	797 (16.20)
**Total**	**2,371 (100)**	**2,530 (100)**	**20 (100)**	**4,921 (100)**

### Single/multiple suspects

There were 10,040 unique crimes; 3,304 (32.91%) had no identified suspect, leaving 6,736 unique crimes where a suspect had been identified. Of these, 211 (3.13%) had multiple suspect IDs associated with them. The number of identified suspects per incident ranged from 1 to 10. There were also 107 (1.07%) occurrences which were classified as being conducted by multiple undefined offenders (this was indicated in the offence description and only had one suspect ID associated with them), bringing the total number of offences committed by multiple suspects to 318. The 107 offenders listed as “multiple undefined” are represented in Table 8 under “multiple unknown” because it is unknown how many suspects were involved in these offences.

**TABLE 8:** The number of offences committed by single versus multiple suspects.

	Rape	SSO	Non-contact	Total
1 suspect	3,132 (94.17)	3,269 (96.37)	17 (94.44)	6,418 (95.28)
2 suspects	68 (2.04)	98 (2.89)	1 (5.56)	167 (2.48)
3+ suspects	19 (0.57)	25 (0.74)	–	44 (0.65)
Unknown multiple suspects	107 (3.22)	–	–	107 (1.59)
**Total**	**3,326 (100)**	**3,392 (100)**	**18 (100)**	**6,736 (100)**

### Outcomes

There were 880 (8.50%) cases where the outcome was missing (likely due to the fact that these cases were still ongoing). Where outcome was present, the majority of cases were either NFAed or the victim decided they did not want to proceed with the investigation, as shown in Table 9. Differences between offence type by outcome were significant (*x*^2^ = 305.35, *p* < 0.001). SSO cases were more likely to be closed due to logistical reasons or be resolved but not charged than rape cases. Non-contact cases were more likely than rape and SSO to end in NFA, likewise, SSO was more likely than rape to end in NFA. Rape cases were least likely to end in a charge and most likely to end in an outcome of victim does not want to proceed, whilst non-contact cases were least likely to end in an outcome of victim does not want to proceed.

**TABLE 9:** The charges associated with each offence, broken down by offence type.

	Rape	SSO	Non-contact	Total
Logistical reasons	34 (0.76)	75 (1.51)	1 (3.70)	110 (1.16)
NFA	1,666 (37.25)	2,107 (42.41)	18 (66.67)	3,791 (40.04)
Resolved but not charged	42 (0.94)	216 (4.35)	1 (3.70)	259 (2.74)
Charged	117 (2.62)	325 (6.54)	4 (14.81)	446 (4.71)
Victim does not want to proceed	2,614 (58.44)	2,245 (45.19)	3 (11.11)	4,862 (51.35)
**Total**	**4,473 (100)**	**4,968 (100)**	**27 (100)**	**9,468 (100)**

### Factors predicting a charge outcome

In the multivariate analysis, type of offence, victim–suspect relationship, and suspect ethnicity predicted a charge outcome (see Table 10). SSO cases had greater odds (OR 3.22; 95% CI 2.06–5.02) of a charge outcome than rape cases. Compared to acquaintances, partners (OR 0.55; 95% CI 0.31–0.97) and relatives (OR 0.41; 95% CI 0.17–0.98) had decreased odds of having a charge outcome, whereas strangers had increased odds (OR 2.59; 95% CI 1.64–4.10), and dark European suspects (OR 4.00; 95% CI 1.89–8.47) and Arab or North African suspects (OR 6.86; 95% CI 1.61–29.23) had greater odds of a charge outcome than white suspects. The relationship for Black suspects just missed significance (*p* = 0.051). However, the model only explained 14.7% of the variance.

**TABLE 10:** Univariate and multivariate binary logistic regression predicting a charge outcome.

	Univariate logistic regression analysis			Multiple logistic regression analysis^a^		
		
Factor	OR	(95% CIs)	*P*-value	OR	(95% CIs)	*P*-value
**Offence type**						
Rape	1			1		
SSO	2.67	2.16–3.31	<0.001	3.22	2.06–5.02	<0.001
Non-contact	5.52	1.91–15.91	0.002	0.00	0.00	1.00
**Relationship**						
Acquaintance	1			1		
Relative	1.24	0.82–1.88	0.316	0.41	0.17–0.98	0.045
Partner	0.49	0.30–0.81	0.005	0.55	0.31–0.97	0.04
Stranger	2.10	1.46–3.02	<0.001	2.59	1.64–4.10	<0.001
**Victim gender**				–		
Male	1			–		
Female	1.02	0.77–1.35	0.92	–		
**Suspect gender**				–		
Male	1			–		
Female	0.53	0.29–0.97	0.04	–		
**Victim age** ^b^	0.98	0.98–99	<0.001	–		
**Suspect age** ^b^	1.01	1.01–1.02	<0.001	–		
**Victim ethnicity**				–		
IC1 – White	1			–		
IC2 – Dark European	0.83	0.30–2.26	0.71	–		
IC3 – Black	0.48	0.18–1.29	0.14	–		
IC4 – Asian	1.12	0.41–3.06	0.83	–		
IC5 – Chinese, Japanese, and other Southeast Asian	0.00		0.99	–		
IC6 – Arab or North African	0.00		0.99	–		
Other	0.94	0.41–2.16	0.89	–		
**Suspect ethnicity**						
IC1 – White	1			1		
IC2 – Dark European	2.38	1.38–4.11	0.002	4.00	1.89–8.47	<0.001
IC3 – Black	1.94	1.37–2.76	<0.001	1.78	1.00–3.19	0.051
IC4 – Asian	1.07	0.57–2.01	0.83	1.50	0.57–3.98	0.412
IC5 – Chinese, Japanese, and other Southeast Asian	1.63	0.21–12.96	0.64	0.00	0.00	0.999
IC6 – Arab or North African	5.29	2.44–11.49	<0.001	6.86	1.61–29.23	0.009
Other	1.68	0.83–3.39	0.15	1.32	0.39–4.46	0.658

^a^Variables included offence type, relationship, suspect sex, victim age, suspect age, and suspect ethnicity.

^b^At time of the offence.

For the outcome victim does not want to proceed, the final model included type of offence, victim–suspect relationship, and victim ethnicity (see Table 11). SSO cases had decreased odds (OR 0.72; 95% CI 0.61–0.86) of receiving an outcome of victim does not want to proceed. Compared to acquaintances, partners had increased odds (OR 1.50; 95% CI 1.21–1.86) of an outcome of victim does not want to proceed while strangers had decreased odds of this outcome (OR 0.60; 95% CI 0.47–0.76), and victims with the ethnicity “other” had increased odds (OR 2.03; 95% CI 1.18–3.49) of an outcome of victim does not want to proceed compared to white victims. However, the model only explained 5.1% of the variance.

**TABLE 11:** Univariate and multivariate binary logistic regression predicting an outcome of victim does not want to proceed.

	Univariate logistic regression analysis			Multiple logistic regression analysis^a^		
		
Factor	OR	(95% CIs)	*P*-value	OR	(95% CIs)	*P*-value
**Offence type**						
Rape	1			1		
SSO	0.59	0.54–0.64	<0.001	0.72	0.61–0.86	<0.001
Non-contact	0.09	0.03–0.30	<0.001	0.29	0.03–2.80	0.283
**Relationship**						
Acquaintance	1			1		
Relative	0.69	0.58–0.82	<0.001	0.92	0.71–1.21	0.559
Partner	1.71	1.46–1.99	<0.001	1.50	1.21–1.86	<0.001
Stranger	0.58	0.49–0.69	<0.001	0.60	0.47–0.76	<0.001
**Victim gender**				–		
Male	1			–		
Female	1.42	1.26–1.60	<0.001	–		
**Suspect gender**				–		
Male	1			–		
Female	1.08	0.86–1.37	0.504	–		
**Victim age** ^b^	1.01	1.01–1.01	<0.001	–		
**Suspect age** ^b^	0.999	0.99–1.00	0.588	–		
**Victim ethnicity**						
IC1 – White	1			1		
IC2 – Dark European	0.86	0.61–1.23	0.42	0.73	0.43–1.24	0.24
IC3 – Black	1.47	1.11–1.96	0.007	1.45	0.86–2.42	0.16
IC4 – Asian	1.07	0.70–1.62	0.768	0.69	0.37–1.29	0.25
IC5 – Chinese, Japanese, and other Southeast Asian	0.80	0.33–1.92	0.616	0.48	0.04–5.27	0.55
IC6 – Arab or North African	1.12	0.35–3.53	0.849	0.96	0.09–10.73	0.974
Other	1.74	1.24–2.42	0.001	2.03	1.18–3.49	0.010
**Suspect ethnicity**				–		
IC1 – White	1			–		
IC2 – Dark European	0.68	0.46–1.01	0.054	–		
IC3 – Black	1.10	0.87–1.38	0.419	–		
IC4 – Asian	0.97	0.69–1.37	0.868	–		
IC5 – Chinese, Japanese, and other Southeast Asian	0.51	0.13–2.03	0.338	–		
IC6 – Arab or North African	1.02	0.47–2.20	0.970	–		
Other	0.94	0.60–1.47	0.788	–		

^a^Variables included offence type, relationship, victim sex, victim age, and victim ethnicity.

^b^At time of the offence.

## Discussion

### What the profile of rape and serious sexual offences and reporting rates look like in Avon and Somerset Constabulary

This dataset allowed us to gain an insight into the victim, suspect, and types of offences reported to ASC over a 3-year period. The sample comprised of just under 50% rape offences and just over 50% SSOs, with non-contact RASSO (which, as noted in Supplementary Appendix A, does not include offences such as exhibitionism or voyeurism), accounting for just 0.3% of the sample. The definition of RASSO was decided on by ASC, and these non-contact RASSO cases seem to conceptualised as offences that led to a rape or SSO, despite the offender themselves not committing the contact offence. The average age of the victims at the time of the offences was 24.66 years, with SSO offenders younger than rape offenders, and non-contact offenders younger than rape and SSO offenders. The majority of victims were female (86.41%), with the split less pronounced for non-contact offences (male = 34.38%; female = 65.63%). The majority of victims were white. In around two thirds of the incidents (67.95%) at least one suspect had been identified. When this was the case, the average offender age was 32.59 years old, and 95.53% offenders were male, with this gender split remaining relatively consistent across all crime types. As with the offenders, the majority of suspects were white. In terms of the victim–suspect relationship, and in line with previous research, the most common relationship was acquaintance, while almost a quarter of offences were committed by partners or ex-partners.

It is important to caveat this profile of RASSO with the fact that these are the offences that are reported to the police, so while this may provide us with a more complete picture of sex offending, including where suspects have been named in a police investigation, this will still necessarily omit offences that have not been reported. As noted above, there are likely to be factors that affect reporting rates, such as the suspect–victim relationship, so this needs to be considered when taking these figures into account, particularly when considering that only around 11% of rape offences are actually reported in the first place (Stern, 2010).

It is also important to highlight that there are significant amounts of missing data in our dataset, such as the 52.33% of missing suspect ethnicity (even when the suspect was identified) or the 52.44% of missing suspect–victim relationship data. It is not possible to know whether the types of offence, suspect, or victim involved affects a police officer’s decision to record this information. However, the fact that 32.05% of suspects were not identified, but only 16.20% of offenders were strangers (not including where data were missing), suggests that even when victim–suspect relationship is known it is not being recorded. This may be indication of instances where relationship type – particularly where they are acquaintances, relatives, or partners/ex-partners – are not being systematically recorded.

Most of the cases had policing outcomes attributed to them; however, 8.50% did not, even though the data were recorded between January 2018 and December 2020, but not compiled until January 2021. This means that these 8.50% of cases with outstanding outcomes were at least 1 year old from the date of reporting. This is in line with current figures demonstrating the lengthy nature of RASSO investigations (Murray, 2022). Where outcomes were available, the majority of cases were either dropped by the police or the victim decided not to proceed. Only in 4.71% of offences was there a charge outcome, which dropped to 2.62% for rape (the charge rates of SSO and non-contact rates were higher at 6.54% and 14.81%, respectively). Again, this is in line with recent figures which demonstrates the low number of charges overall and the high level of victim attrition (Ministry of Justice, 2021).

### Are there any factors associated with charges/victim declines to prosecute?

In terms of the factors that predicted a charge outcome, strangers were more likely and relatives/partners and ex-partners were less likely to have charge outcomes than other types of victim–suspect relationships, and SSO cases were more likely to be charged than rape offences. The fact that stranger offences are more likely to be charged is in line with the literature discussed above (e.g., Rumney et al., 2016), and with the idea that these offences are more likely to be taken seriously and thus progressed through the criminal justice system. In terms of the SSO cases being more likely to result in a charge, we could hypothesise that this is due to the victims in these cases being significantly younger, and therefore less likely to be subject to victim blaming or rape myth stereotyping. However, age was not part of the model that predicts charge outcomes so this theory requires further exploration.

There were also several factors that predicted victim attrition from the investigation process. SSO cases, while being more likely to predict charge outcomes than rape offences, were also less likely to have an outcome of victim does not want to proceed. Victims where the suspect in the case was either a partner or an ex-partner were more likely not to proceed with the investigation than acquaintances, which is a particular concern from a safeguarding perspective where victims may well still be living with the perpetrator. Offences involving strangers were also less likely to have an outcome of victim does not want to proceed, again suggesting these offences are taken more seriously and therefore the victim feels more able to proceed through the process. It is important to note here that both of these regression models – particularly the model predicting victim attrition – explained only a small amount of the variance, so any conclusions drawn from these analyses should be treated with caution.

While some of these findings suggest that there may be aspects of rape stereotyping that is feeding into the investigative process and ultimately the policing outcomes assigned to cases, there were also some other factors predictive of charges and victim attrition that may be indicative of other types of bias within policing. Cases with some non-white suspects were more likely to result in a charge, which potentially demonstrates a worrying increase in the targeting of non-white suspects. Previous research has demonstrated that white women were more likely to have their cases prosecuted than women from ethnic minorities (Campbell et al., 2001), while Hohl and Stanko (2015) determined that that non-white suspects’ cases were less likely to be “no-crimed.” Taken together, this and previous research raise concerns of the influence of stereotypes around victims and suspects on police decision making.

The discrepancy we see in outcomes may be related to the fact that some offences are inherently more difficult to investigate. For instance, are domestic offences more likely to take place within a home location and without witnesses, compared to stranger offences that may take place in public spaces? While there is some limited information which tells us about the specific behavioural characteristics of offences where relationship type or suspect/victim characteristics differ (e.g., more weapon threat seen in stranger compared to acquaintance rape; Bownes et al., 1991) there is little research in this area which does not provide us with a comprehensive views of these differences, and this dataset did not provide this level of detail. However, perhaps the more important question to ask here is whether the degree of investigative difficulty, whether perceived or real, is a hindrance to a full investigation being conducted in the first place. The fact that research and recent reviews all suggest that a victim’s credibility is under scrutiny over and above the circumstances of the offence or the suspect identified, suggests that law enforcement are not taking a suspect focused approach to the investigation which may be hindering them from considering potential lines of inquiry when the investigation is perceived to be difficult. As noted above, while specialism in RASSO investigations may prove useful in many contexts (Dalton et al., 2022), including specialist training designed to combat rape myth acceptance, real attitudinal and cognitive change seems to be difficult to achieve (Lonsway et al., 2001). In short, it is possible that a combination of victim credibility and factors that fall outside of the “real rape” scenario are preventing thorough and fair investigations from being conducted. This lack of willingness to fully investigate these types of offences, particularly where it is assumed that the allegation turns on an issue of consent (not that this, in itself, means that a case cannot be progressed), means that law enforcement may well be missing potential opportunities to target repeat and persistent offending often demonstrated by sex offenders (Abel et al., 1987).

### Limitations and future work

This is a preliminary study into the links between victim, suspect, and offence characteristics and the associated police outcomes, based on data from one police force. Further work needs to be conducted to see if the trends seen here are indicative of trends nationally. More information also needs to be gathered on the way in which victim, suspect, and offence characteristics have an impact on, not only the ultimate policing outcome, but also the different aspects of the investigation. This includes the efforts involved to investigate reasonable lines of enquiry and the timeliness with which this is conducted, the point at which the suspect is spoken to, and the manner in which the victim is treated. Further work is also required to explore the particular circumstances of the offences, such as the type of approach used by the suspect to contact the victim, and the method of control used, to assess how these factors affect the investigation and associated outcomes. For this, and as noted above, information needs to be routinely gathered by law enforcement, not least so that they can conduct their own analyses into the efficacy of their procedures. Importantly, where iniquities in the investigative process are found, these need to be eradicated. Work with law enforcement is required to understand where biases in conducting investigations occur and why, in order that they can be eliminated. Equally, work should also be conducted to examine the effects of the groupings used here, including the victim–suspect relationship groups used and the age ranges chosen. Further work could also be conducted to explore the differences between different types of suspects and victims where more detailed information, such as different types of acquaintances, for instance, are recorded in different police forces. The consistency with which these types of data are recorded by police is also a necessary avenue of future research, to assess the variance with which these variables are recorded and consequently to understand how this may affect the results of studies such as these. The amount of missing data seen here suggests that data recording may not be a straightforward (or even routine, for some variables) process in policing.

## Conclusion

Many people become victims of RASSO in their lifetime, and yet the police response to these types of crimes is inadequate, with many victims feeling unable to report to the police, and when they do, being met with unequal treatment and low rates of charge. This study confirmed these low charge rates, as well as high victim attrition rates, in our 3-year sample. The analyses also demonstrated inequalities in outcomes, depending on offence, victim, and suspect type. These findings are in line with previous research and highlight the urgent need for improvement to our criminal justice system process to provide victims with better access to justice.

## Data availability statement

The data analysed in this study was subject to the following licences/restrictions: the dataset is subject to agreements with the data owners that prohibit the researchers from sharing it. Requests to access these datasets should be directed to Avon and Somerset Constabulary.

## Author contributions

KD and MH: concept, writing, editing, and supervision. RS: analysis, writing, and editing. EC and MC: writing. All authors contributed to the article and approved the submitted version.

## Footnotes

1 We have used the term “victim” to reflect the terminology used in policing and how the data were recorded in this dataset which is the primary focus of this paper. This is not to ignore or negate the significance of the use of different terminology in this field and the alternatives such as “survivor” and “victim-survivor.”

2 Operation Soteria Bluestone is a United Kingdom Home Office-funded programme designed to improve the investigation of rape and serious sexual offences (RASSO) in England and Wales. It is a unique project which is underpinned by rigorous social science. With multi-disciplined academics located in multiple universities, mixed qualitative and quantitative methods are applied to a five pillared approach to organisational change with police forces, uplifting the capability of more specialist police decision-making in RASSO cases. The research informs policing practice as well as government policy and is set to inform a national change. These research informed pillars pinpoint specific areas for improvement which will form part of the new framework for investigating RASSO: (1) suspect-focused investigations; (2) disrupting repeat offenders; (3) victim engagement as procedural justice; (4) promoting better learning, development, and wellbeing for police officers; and (5) using data more effectively in RASSO investigations. The pathfinder project started in 2021, based in Avon and Somerset Constabulary. Designed by Katrin Hohl and Betsy Stanko, the pillar leads include Kari Davies, Miranda Horvath, Kelly Johnson, Jo Lovett, Olivia Smith, and Emma Williams.

3 This excluded 277 duplicate cases, identified when they had the same offender, victim, and offence ID.

4 The term suspect has been used in recognition of the fact that those named have not necessarily been charged and convicted for the offence in which they were named.

5 We followed ASC’s definitions of these categories in order to group the more specific variables. All partner and ex-partner variables were grouped into “partner,” and all types of family and relative variables (including step-relatives) were categorised into “relative.” The “stranger” category contains only the “stranger” variable, defined as both suspects who had had no contact with the victim prior to the offence, as well as those suspects who had had limited contact with the victim which may have facilitated the offence (e.g., meeting on a dating app). The “acquaintance” category, again, only contains the “acquaintance” variable, defined in ASC as any suspects not falling into the above categories, and ranging from suspects the victim may have known casually, friends, colleagues, and people in positions of power such as teachers.

6 The age ranges were chosen as a reflection of the offence categories used in England and Wales legislation.
