# Supplementary material for: Understanding sexual violence and factors related to police outcomes
Source: Front Psychol. 2022 Sep 1;13:977318. doi: 10.3389/fpsyg.2022.977318 (PMC9477140; doi:10.3389/fpsyg.2022.977318)
Supplement: Supplementary file 1 [file Data_Sheet_1.docx]

# Appendices

***Appendix A – List of RASSO offences included in the dataset***

*Rape offence*

- Rape a woman 16 years of age or over - SOA 2003 (recordable)
- Rape of a female aged 16 or over
- Attempted rape of a female aged 16 or over
- Rape a girl aged 13 / 14 / 15 - SOA 2003 (recordable)
- Rape of a female aged under 16; Rape of a female aged under 16
- Attempted rape of a female aged under 16
- Rape of a female child under 13 by a male
- Attempted rape of a female child under 13 by a male
- Rape of a male aged 16 or over
- Attempted rape of a male aged 16 or over
- Rape of a male aged under 16
- Attempted rape of a male aged under 16
- Rape of a male child under 13 by a male
- Attempted rape of a male child under 13 by a male
- Rape of a female aged under 16 - multiple undefined offenders
- Rape a woman 16 years of age or over - multiple undefined offenders
- Attempted rape of a female aged 16 or over - multiple undefined offenders
- Rape of a female child under 13 by a male - multiple undefined offenders
- Rape of a male aged under 16 - multiple undefined offenders
- Rape of a male aged 16 or over - multiple undefined offenders
- Rape of a male child under 13 by a male - multiple undefined offenders

*Sexual assault offence*

- Assault on a female by penetration
- Attempt to sexually assault by penetration a female aged 13 and over (recordable)
- Conspire to sexually assault a female person 13 or over by penetration (recordable)
- Attempt sexual assault on a female - SOA 2003 (recordable)
- Sexual assault on a female
- Assault of a female child under 13 by penetration
- Attempt to assault a girl under 13 by penetration with a part of your body / a thing (recordable)
- Aid abet the sexual assault of a female child under 13 by touching (recordable)
- Attempt to sexually assault a girl under 13 by touching (recordable)
- Sexual assault of a female child under 13
- Attempt to cause / incite a girl under 13 to engage in sexual activity - penetration (recordable)
- Causing or inciting a child under 13 to engage in sexual activity - Female child – penetration
- Conspire to cause / incite a girl under 13 to engage in a penetrative sexual activity (recordable)
- Attempt to cause / incite a female child aged under 13 to engage in sexual activity - no penetration (recordable)
- Causing or inciting a child under 13 to engage in sexual activity - Female child - no penetration
- Conspire to cause / incite a girl under the age of 13 to engage in sexual activity - no penetration (recordable)
- Causing or inciting a child under 13 to engage in sexual activity - Male child – penetration
- Causing or inciting a child under 13 to engage in sexual activity - Male child - no penetration
- Offender 18 or over engage in penetrative sexual activity with girl under 13 - SOA 2003 (recordable)
- Cause / incite a female child under 13 to engage in sexual activity - offender 18 or over - penetration (recordable)
- Cause / incite a male child under 13 to engage in sexual activity - offender 18 or over - penetration (recordable)
- Attempt to engage in a penetrative sexual activity with a girl under 13 - offender under 18 (recordable)
- Sexual activity with a child under 13 by an offender under 18 years of age - Female child – penetration
- Sexual activity with a child under 13 by an offender under 18 years of age - Male child – penetration
- Causing or inciting a child under 13 to engage in sexual activity by an offender under 18 years of age - Female child – penetration
- Causing or inciting a child under 13 to engage in sexual activity by an offender under 18 years of age - Male child – penetration
- Offender 18 or over engage in non penetrative sexual activity with girl under 13 - SOA 2003 (recordable)
- Offender 18 or over cause / incite a girl under 13 to engage in sexual activity - no penetration - SOA 2003 (recordable)
- Offender 18 or over attempt to cause / incite a boy under 13 to engage in sexual activity - no penetration (recordable)
- Offender 18 or over cause / incite a boy under 13 to engage in sexual activity - no penetration (recordable)
- Sexual activity with a child under 13 by an offender under 18 years of age - Female child - no penetration
- Sexual activity with a child under 13 by an offender under 18 years of age - Male child - no penetration
- Causing or inciting a child under 13 to engage in sexual activity by an offender under 18 years of age - Female child - no penetration
- Causing or inciting a child under 13 to engage in sexual activity by an offender under 18 years of age - Male child - no penetration
- Causing a person to engage in sexual activity without consent - Female person
- Causing a person to engage in sexual activity without consent - Male person
- Causing a person to engage in sexual activity without consent - Female person - no penetration
- Conspire to cause a female 13 or over to engage in sexual activity - no penetration (recordable)
- Causing a person to engage in sexual activity without consent - Male person - no penetration
- Sexual activity with a person with a mental disorder impeding choice - Male person
- Sexual activity with a person with a mental disorder impeding choice - Female person
- Sexual activity with a person with a mental disorder impeding choice - Male person - no penetration
- Sexual activity with a person with a mental disorder impeding choice - Female person - no penetration
- Causing or inciting a person with a mental disorder impeding choice to engage in sexual activity - Male person
- Causing or inciting a person with a mental disorder impeding choice to engage in sexual activity - Female person
- Causing or inciting a person with a mental disorder impeding choice to engage in sexual activity - Male person - no penetration
- Causing or inciting a person with a mental disorder impeding choice to engage in sexual activity - Female person - no penetration
- Inducement, threat or deception to procure sexual activity with a person with a mental disorder - no penetration
- Causing a person with a mental disorder to engage in sexual activity by inducement, threat or deception – penetration
- Causing a person with a mental disorder to engage in sexual activity by inducement, threat or deception - no penetration
- Care workers: Sexual activity with a person with a mental disorder - Male person
- Care workers: Sexual activity with a person with a mental disorder - Female person
- Care workers: Sexual activity with a person with a mental disorder - Male person - no penetration
- Care workers: Sexual activity with a person with a mental disorder - Female person - no penetration
- Care workers: Causing or inciting sexual activity (person with mental disorder) – Penetration
- Care workers: Causing or inciting sexual activity (person with mental disorder) - No penetration
- Arranging or facilitating the commission of a child sex offence
- Paying for the sexual services of a child - Male child under 13
- Paying for the sexual services of a child - Female child under 18
- Paying for the sexual services of a child - Male child under 18
- Attempt to cause / incite the sexual exploitation of a child aged 13 - 17 (recordable)
- Causing or inciting child prostitution or pornography - Child 13 – 17
- Causing or inciting child prostitution or pornography - Child 13 – 17
- Controlling a child prostitute or a child involved in pornography - Child 13 – 17
- Arranging or facilitating child prostitution or pornography - Child 13 – 17 (recordable)
- Arranging or facilitating child prostitution or pornography - Child 13 – 17
- Causing or inciting child prostitution or pornography - Child under 13
- Controlling a child prostitute or a child involved in pornography - Child under 13
- Arranging or facilitating child prostitution or pornography - Child under 13
- Paying for the sexual services of a child - Female child under 16
- Trafficking persons into the United Kingdom for sexual exploitation (recordable)
- Trafficking persons within the United Kingdom for sexual exploitation (recordable)

*Non-contact offence*

- Engage in sexual activity in presence of a child under 13 - offender 18 or over (recordable)
- Engaging in sexual activity in the presence of a child under 13 by an offender over 18 years of age
- Causing a child under 13 to watch a sexual act by an offender over 18 years of age
- Offender 18 or over cause a child under 13 to watch / look at an image of sexual activity - SOA 2003 (recordable)
- Engaging in sexual activity in the presence of a child under 13 by an offender under 18 years of age
- Causing a child under 13 to watch a sexual act by an offender under 18 years of age
- Care workers: Sexual activity in the presence of a person with a mental disorder
- Care workers: Causing a person with a mental disorder or learning disability to watch a sexual act

***Appendix B – full list of relationship categories***

Acquaintance

Brother (incl. step/half/adopted)

Daughter (incl. step/half/adopted)

Ex-partner: Heterosexual

Ex-partner: Non-heterosexual

Ex-partner: Not known

Grandchild (incl. step/adopted)

Grandparent (incl. step/adopted)

Not known/No current suspect

Not seen by victim

Other relative

Other sexual relationship

Parent (incl. step/adopted)

Partner: Heterosexual

Partner: Non-heterosexual

Partner: Not known

Sister (incl. step/half/adopted)

Son (incl. step/half/adopted)

Stranger

Victimless/crime against state

***Appendix C – full list of policing outcomes***

Filed - Incident Report OR CRI

New - Under Investigation

OC01 - Charge/Summons

OC02 - Caution - Youths

OC03 - Caution - Adults

OC04 - TIC

OC05 - Offender Dead

OC08 - Community Resolution

OC09 - Not in Public Interest (CPS)

OC10 - Not in Public Interest (Pol)

OC11 - Offender Under Age

OC12 - Offender too ill

OC13 - Victim/Witness Dead/Ill

OC14 - No Sus/Victim Decline

OC15 - Sus/Insufficient.Evidence

OC16 - Sus/Victim Declines

OC17 - Time Limit Expired

OC18 - No Suspect/No Line Enquiry/Filed

OC20 – Transferred Other Agency

OC21 - Investigation not in Public Interest

OC22 - Diversionary, educational
